# Pancreaticoduodenectomy: Volume is not Associated with Outcome within an Academic Health Care System

**DOI:** 10.1155/2008/825940

**Published:** 2008-01-29

**Authors:** Micheal T. Schell, Anthony Barcia, Austin L. Spitzer, Hobart W. Harris

**Affiliations:** ^1^Department of Surgery, University of California, 513 Parnassus Avenue, S-320 San Francisco, CA 94143-0104, USA; ^2^California State University, East Bay, 25800 Carlos Bee Boulevard, Hayward, CA 94542, USA

## Abstract

*Hypothesis*. Smaller and lower-volume hospitals can attain surgical outcomes similar to high-volume centers if they incorporate the expertise and health care pathways of high-volume centers. *Setting*. The academic tertiary care center, Moffit-Long Hospital (ML); the community-based Mount Zion Hospital (ZION); the San Francisco County General Hospital (SFGH); and the Veterans Affairs Medical Center of San Francisco (VAMC). *Patients*. 369 patients who underwent pancreaticoduodenectomy between October 1989 and June 2003 at the University of California, San Francisco (UCSF) affiliated hospitals. *Interventions*. Pancreaticoduodenectomy. *Design*. Retrospective chart review. To correct for the potentially confounding effect of small case volumes and event rates, data for SFGH, VAMC, and ZION was combined (Small Volume Hospital Group; SVHG) and compared against data for ML. *Main Outcome Measures*. Complication rates; three-year and five-year survival rates. *Results*. The average patient age and health, as determined by ASA score, were similar between ML and the SVHG. The postoperative complication rate did not differ significantly between ML and the SVGH (58.8% versus 63.1%). Patients that experienced a complication averaged 2.5 complications in both groups. The perioperative mortality rate was 4% for patients undergoing pancreaticoduodenectomy at either ML or the SVGH. Although the 3-year survival rate for patients with adenocarcinoma of the pancreas was nearly twice as high at ML (31.2% versus 18.3% at SVHG), there was no significant difference in the 5-year survival rates (19% at ML versus 18.3% at SVHG). *Conclusions*. Low-volume hospitals can achieve similar outcomes to high-volume tertiary care centers provided they import the expertise and care pathways necessary for improved results.

## 1. INTRODUCTION

Pancreaticoduodenectomy is the only potentially curative treatment for pancreatic cancer, which ranks fifth in cancer-related mortality worldwide [1–4]. However, because pancreatic cancer usually presents late, only 10% to 20% of patients are candidates for pancreaticoduodenectomy [5, 6], a potentially lifesaving procedure that is associated with high morbidity and a disappointing 5-year survival rate of 10% to 29% [7–12].

Evidence showing better outcomes after complex surgical procedures such as pancreaticoduodenectomy in high-volume hospitals has led to the suggestion that these procedures are regionalized to high-volume institutions [13–17]. However, although better outcomes have been attributed to a high volume of pancreaticoduodenectomies specifically [18–20], some believe that it is a high volume of complex procedures performed at a high-volume hospital, and not necessarily a specific procedure itself that is responsible for better outcomes [21]. We also believe that it is the high volume of complex procedures performed at academic tertiary referral centers that enables them to optimize their operative and perioperative health care delivery systems to achieve better outcomes. We, therefore, hypothesized that if the optimized health care delivery systems of high-volume hospitals were exported to smaller, low-volume hospitals, their outcomes would approximate those of larger, high-volume hospitals. To examine this hypothesis, we retrospectively analyzed the surgical and survival outcomes for patients treated with pancreaticoduodenectomy at the low- and high-volume hospitals affiliated with the University of California, San Francisco.

## 2. METHODS

### 2.1. Data source

This was a retrospective chart review of 369 patients treated within the hospitals affiliated with the University of California, San Francisco (UCSF) from October 1989 to June 2003. The hospitals include the academic tertiary care center, Moffit-Long Hospital (ML, n=301), which averaged 23 pancreaticoduodenectomies per year; the Veterans Affairs Medical Center of San Francisco (VAMC, n=41), which averaged 3 pancreaticoduodenectomies per year; and the community-based Mount Zion Hospital (ZION, n=9) and the San Francisco County General Hospital (SFGH, n=18), each of which averaged approximately 1 pancreaticoduodenectomy per year. Patients were identified by using discharge codes for pancreatectomy (52.51, 52.53, 52.6, 52.7) from the International Classification of Diseases, Ninth Revision, Clinical Modification (ICD-9), and Current Procedural Terminology (CPT) codes for the Whipple-type procedure with pancreaticojejunostomy (48150) and without pancreaticojejunostomy (48152), the pylorus-sparing Whipple-type procedure with pancreatojejunostomy (48153) and without pancreatojejunostomy (48154), as well as total pancreatectomy (48155). Followup data was obtained through the cancer registry of each institution. At the time of the study, 184 patients had died, 9 were lost-to-followup, and the remaining 176 had been monitored for an average of 5.0±3.7 years (mean ± SD). This study was approved by the UCSF Committee on Human Research and by the individual Institutional Review Boards at all hospitals where applicable.

### 2.2. Outcome variables

Patient demographics and relevant patient history including age, sex, date of birth, race, and co-morbidities were documented. Inpatient variables included the date of procedure, complications, length of stay in the intensive care unit (ICU) and hospital, and disposition after discharge. We also recorded data regarding the indication for surgery, the American Society of Anesthesiologists (ASA) risk score, the type of resection (pylorus- preserving versus classic; distal gastrectomy), the extent of pancreatic resection, whether the superior mesenteric vein was resected, intraoperative blood loss, the incidence and number of blood transfusions, and operative time. Pathology data consisted of tumor site of origin, tumor differentiation and diameter, resection margins, and evidence of perineural or vascular invasion. Perioperative mortality (defined as death in hospital or within 30 days of discharge) and long-term survival were ascertained for patients seen at the four hospitals.

### 2.3. Statistical analysis

The Kruskal-Wallis test was used to compare the mean and median values of continuous data. Fisher's exact test was used to evaluate statistical significance for categorical data. Significance was set at P≤.05. The Kaplan-Meier method was used to calculate three-year and five-year survival curves, which were compared using the log-rank test. To correct for the potentially confounding effect of small case volumes and event rates, data for SFGH, VAMC, and ZION was combined (Low Volume Hospital Group; LVHG) and compared against data for ML.

## 3. RESULTS

**Figure 1:**
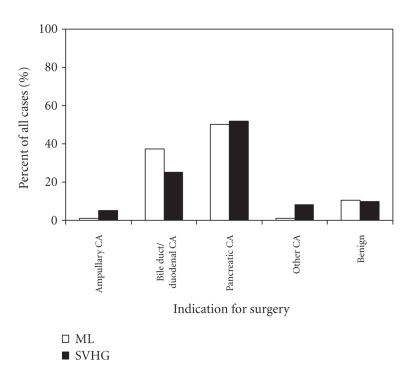
Indication for pancreaticoduodenectomy at UCSF-affiliated hospitals, 1989–2003. ML, Moffit-Long Hospital; LVHG, low-volume hospital group, is the combined data for San Francisco County General Hospital, Veterans Affairs Medical Center of San Francisco, and Mount Zion Hospital (P=.003 by Fisher's exact test).

The average patient age and health, as determined by ASA score, were similar, though the sex and race of the patient populations were significantly different between ML and the LVHG (Table 1). Although the most common indication for surgery at ML (50.2%) and LVHG (51.7%) was pancreatic cancer (Figure 1), there was significant variation between the groups. Pylorus-preserving procedures were performed more often than classic distal gastrectomy procedures both at ML (64.7% versus 35.3%) and LVHG (56.1% versus 43.9%) without a significant difference in the rates (Table 2). Superior mesenteric vein resection was performed in 9% of patients in both groups. Although average blood loss differed significantly between ML and LVHG, the transfusion rate and average number of transfused units of blood in patients requiring transfusion did not. Total operative time and length of stay in the ICU and in the hospital were significantly shorter at ML than at the LVHG (Table 2).

The postoperative complication rate did not differ significantly between ML and the SVHG (58.8% versus 63.1%, Table 3). The average number of complications per patient, among those that experienced a complication, was 2.5. Only cardiopulmonary complications occurred at a significantly different rate, and were the most frequent complication in each group.

**Figure 2:**
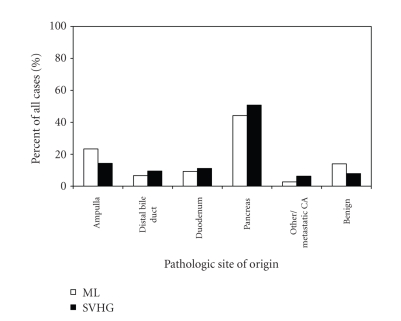
Pathological tumor site for patients undergoing pancreaticoduodenectomy at UCSF-affiliated hospitals, 1989–2003. ML, Moffit-Long Hospital; LVHG, low-volume hospital group, is the combined data for San Francisco County General Hospital, Veterans Affairs Medical Center of San Francisco, and Mount Zion Hospital (P=.201 by Fisher's exact test).

**Figure 3:**
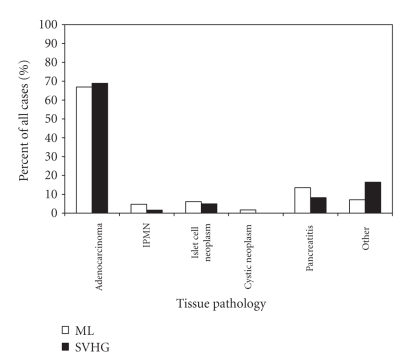
Pathological tumor type for patients undergoing pancreaticoduodenectomy at UCSF-affiliated hospitals, 1989–2003. ML, Moffit-Long Hospital; LVHG, low-volume hospital group, is the combined data for San Francisco County General Hospital, Veterans Affairs Medical Center of San Francisco, and Mount Zion Hospital; IPMN, Intraductal Papillary Mucinous Neoplasm (P=.202 by Fisher's exact test).

The pancreas was the most common site of tumor origin in both groups (Figure 2). Tumor pathology was similar in both groups; adenocarcinoma accounted for approximately 67–69% of the cases (Figure 3). The average tumor diameter, the incidence of positive margins, and the percentages and patterns of patients with a low, moderate, or high grade of tumor differentiation did not differ significantly between LVHG and ML, but neural invasion did (Table 4).

**Figure 4:**
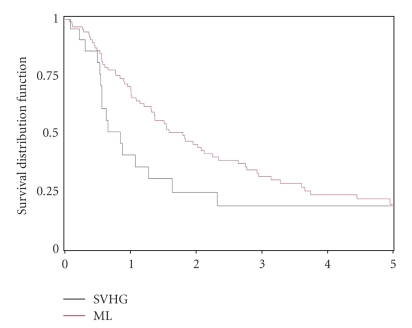
Comparison of Kaplan-Meier survival curves for patients with adenocarcinoma of the pancreas. The survival data from Mount Zion hospital, San Francisco General County hospital, and the Veterans Affairs Medical Center were combined (LVHG) and compared with data from Moffitt-Long Hospital (ML). (P=.035 at 3 years; P=.096 at 5 years, by Log-Rank test).

The perioperative mortality rate for patients undergoing pancreaticoduodenectomy was approximately 4% in both groups (not shown). Although the 3-year survival rate for patients with adenocarcinoma of the pancreas was nearly twice as high at ML (31.2% versus 18.3% at LVHG, Figure 4), there was no significant difference in the 5-year survival rates (19% at ML versus 18.3% at LVHG).

## 4. DISCUSSION

Our results demonstrate that morbidity and mortality outcomes were consistent between the low-volume and high-volume hospitals affiliated with UCSF. We believe that this is due to the sharing of operative techniques and perioperative care pathways, which has enabled the low-volume hospitals to develop a health care delivery system that resembles the large-volume hospital and achieves comparable results.

The low-volume hospital group, which consisted of a community-based hospital and a county general hospital, each of which averaged 1 pancreaticoduodenectomy per year, and a Veterans Affairs Medical Center that averaged 3 pancreaticodudenectomies per year, was able to minimize adverse events to the same extent as the large-volume tertiary center, which averaged 23 pancreaticodudenectomies per year. The hospitals did not differ significantly in the percentage of patients experiencing a complication. In fact, the mean and median numbers of complications among patients who had a complication were exactly the same. Moreover, the pancreatic fistulas and anastomotic leaks that are often associated with longer hospitalizations and increased mortality occurred at statistically equivalent lower rates (12.6 % at ML and 5.9% in the LVHG) than the internationally reported average of 14.3%–26.7% [23]. Uniform patient selection among the hospitals likely aided in the eventual similar outcomes, because although racial and gender differences existed between the LVHG and ML, the general health and age of the patients were similar.

Clinic notes and information obtained through the appropriate cancer registries showed a median followup of 5.0 and 5.9 years at ML and the LVHG, respectively. Although there are inherent limitations to a retrospective chart review, we believe that the prolonged followup period allows us to accurately ascertain the long-term survival through our methods. For all patients who underwent pancreaticoduodenectomy for any indication, perioperative mortality and five-year survival did not differ significantly between the SVGH and ML. In addition, for patients who underwent pancreaticoduodenectomy for adenocarcinoma of the pancreas either at the LVHG or ML, the perioperative mortality and five-year survival rates were similar and comparable to the nationally reported rates of 3%–11% and 10%–29%, respectively [7–12, 22]. However, in our study, as in others, there was a significant difference between the low- and high-volume hospitals in the three-year survival rate for patients treated with pancreaticoduodenectomy for adenocarcinoma of the pancreas [1–4]. Further investigation into postoperative care pathways and procedural capabilities that may account for the early disparity in survival is clearly warranted.

The average operative time in the LVHG was significantly longer than at ML, as was the average blood loss (although only by 96 cc). The clinical importance of an additional blood loss of 96 cc is pedagogical at best, and is further diminished by our finding that the percentage of patients requiring a transfusion and the average number of units transfused in such patients were similar among the groups. The difference in operative time may reflect the advantage that a high volume of cases gives a surgical team in terms of operative efficiency. However, since the rate and number of units transfused, the perioperative mortality, the rate and average number of complications, and the 5-year survival rates were not significantly different between the LVHG and ML, operative efficiency did not seem to affect efficacy.

The greater efficiency in the health care system at ML was also reflected in the significantly shorter stays in both the ICU and the hospital. Although we cannot establish a causal relationship, we believe the longer hospital stays in the LVHG were most likely influenced by the extended ICU stays. Perhaps the inability of the LVHG to provide the necessary care and observation outside the ICU was a factor in the increased length of stay, but further studies are needed to assess whether there is a significant disparity between the technical capabilities of the LVHG and ML. More importantly, if there are important differences in staff or resource capabilities that could be identified and corrected, perhaps the LVHG, as well as other small and low-volume hospitals, could achieve ICU and hospital stays which approximate those of high-volume hospitals.

Optimal long-term surgical outcomes were achieved at the hospitals affiliated with UCSF. For several reasons we believe that these outcomes indicate that health care delivery systems similar to ours can produce comparable results, independent of the volume of pancreaticoduodenectomies performed. First, UCSF residents rotate through all the affiliated hospitals and may transmit or share knowledge gained at the tertiary care center (ML) on how to preoperatively evaluate patients, assist at and perform a pancreaticoduodenectomy, and recognize potential postoperative complications. Secondly, the affiliated hospitals all participate in the UCSF morbidity and mortality education system, which serves to enlighten the surgeons and the ancillary services such as anesthesia, interventional radiology, and nursing. Lastly, expert surgeons and case-related specialists often travel between hospitals to aid in preoperative assessment and the technical aspects of the cases. We consider the low-volume hospitals to be independent institutions that are capable of importing the critical elements of a successful health care delivery system in order to increase the health care options of patients in their surrounding communities. Perhaps the best way to improve access to and the outcomes of complex surgical procedures for the majority of the population in the United States is to develop specific programs in selected low-volume centers that would be affiliated with, modeled after, and thus effectively emulate the practices and processes evident in high-volume hospitals. Despite the intuitive appeal of regionalizing the performance of complex surgical procedures, there are several barriers, including patient preference, that limit the practical utility of this approach. Still, one cannot overemphasize the essential importance of low volume hospitals implementing detailed, comprehensive programs in order to safely provide high-quality, complex surgical services. By identifying, exporting, and implementing the operative decision making and perioperative care pathways that enable high-volume centers to achieve consistently good outcomes into small and low-volume hospitals, their outcomes should approximate those of the high-volume centers. We are encouraged by our early experiences achieving just such a goal in a local, county medical center in Northern California [22].

## Figures and Tables

**Table 1:** Characteristics of patients who underwent pancreaticoduodenectomy at UCSF-affiliated hospitals, 1989–2003.

Characteristics	ML	LVHG	P value^†^
Age, mean (median), year	61 (64)	62 (62)	.887
Sex, no. (%)^‡^	—	—	<.0001
Male	153 (50.8)	59 (86.8)	—
Race, no. (%)^‡^	—	—	<.0001
Asian	33 (11)	7 (10.4)	—
African american	8 (2.7)	13 (19.4)	—
Hispanic	17 (5.6)	5 (7.5)	—
Caucasian	206 (68.4)	37 (55.2)	—
Other	37 (12.3)	5 (7.5)	—
ASA Rating, no. (%)^‡^	—	—	.605
1	4 (1.3)	1 (3.8)	—
2	142 (47.8)	12 (46.2)	—
3	148 (49.8)	13 (50)	—
4	3 (1)	0 (0)	—

^†^According to the Kruskal-Wallis test for continuous data or Fisher's exact test for categorical data. ^‡^Numbers may not sum to group total because data was not available for all patients.

**Table 2:** Operative and perioperative characteristics of patients who underwent pancreaticoduodenectomy at UCSF-affiliated hospitals, 1989–2003.

Characteristics	ML	LVHG	P value^†^
Procedure type, no. (%)	—	—	.206
Pylorus-preserving	194 (64.7)	37 (56.1)	—
Classic; distal gastrectomy	106 (35.3)	29 (43.9)	—
Vein resection, no. (%)	—	—	1.000
No.	274/301 (91)	52/57 (91.2)	—
Blood loss, mean (SD), ml	1166.7 (1410.7)	1262.7 (836.7)	.012
Patients receiving a transfusion, no. (%)	134/301 (44.5)	29/53 (54.7)	.1813
Units transfused, mean (SD)	3.3 (3.9)	2.6 (1.3)	.930
Operative time, mean (SD), h	6.7 (2)	8.3 (2.1)	<.0001
ICU stay, mean (SD), days	2.1 (7)	8.8 (21.6)	<.0001
Post-Op hospital stay, mean (SD), days	16.1 (23.5)	24.5 (24)	<.0001

^†^According to the Kruskal-Wallis test for continuous data or Fisher's exact test for categorical data.

**Table 3:** Complications in patients who underwent pancreaticoduodenectomy at UCSF-affiliated hospitals, 1989–2003.

Complications	ML (n=301)	LVHG (n=68)	P value^†^
Complication, no. (%)	177 (58.8)	41 (60.3)	.579
Complications per patient, mean (SD)	2.5 (2.6)	2.5 (1.8)	.221
General types of complications, no. (%)	—	—	—
Cardiopulmonary	52 (17.3)	20 (29.4)	.028
Wound	41 (13.6)	10 (14.7)	.846
Pancreatic leak or fistula	38 (12.6)	4 (5.9)	.140
Secondary procedures	23 (7.6)	8 (11.8)	.330
Postoperative hemorrhage	19 (6.3)	3 (4.4)	.778
Enteric leak, fistula, or stricture	10 (3.3)	1 (1.5)	.697
Delayed gastric emptying	14 (4.7)	4 (5.9)	.754
Reoperation	22 (7.3)	8 (11.8)	.224

^†^According to Fisher's exact test for categorical data.

**Table 4:** Pathologic diagnosis in patients who underwent pancreaticoduodenectomy at UCSF-affiliated hospitals, 1989–2003.

Characteristics	ML	LVHG	P value^†^
Tumor diameter, mean (SD), cm	3.0 (1.5)	2.9 (1.7)	1.000
Tumor differentiation, no. (%)^‡^	—	—	.575
Low	36 (39.1)	7 (30.4)	—
Moderate	46 (50)	12 (52.2)	—
High	10 (10.9)	4 (17.4)	—
Positive tumor margins, no. (%)	25 (26.9)	6 (25)	1.000
Positive vascular invasion, no. (%)	28 (31.1)	7 (58.3)	.102
Positive neural invasion, no. (%)	54 (60)	14 (87.5)	.047

^†^According to the Kruskal-Wallis test for continuous data or Fisher's exact test for categorical data. ^‡^Numbers may not sum to group total because data was not available for all patients.
